# Multilevel analysis of factors associated with perinatal intimate partner violence among postpartum population in Southern Ethiopia

**DOI:** 10.1038/s41598-022-23645-4

**Published:** 2022-11-08

**Authors:** Tafesse Lamaro Abota, Fikre Enqueselassie Gashe, Negussie Deyessa

**Affiliations:** 1 College of Medicine and Health Sciences, Mizan-Tepi University, Mizan-Aman, Ethiopia; 2 School of Public Health, College of Health Sciences, Addis Ababa University, Addis Ababa, Ethiopia

**Keywords:** Public health, Risk factors

## Abstract

Violence around pregnancy is critical in nature and major public health problem worldwide. Thus, the present study aims to determine the extent of perinatal partner violence and to identify its individual and community-level factors among postpartum women in Southern Ethiopia. A total of 1342 postpartum women nested in 38 ‘Kebles’ (clusters) were enumerated using multistage-clustered sampling techniques for multilevel analysis. Different parameters were computed for model comparison and model fitness. The overall prevalence of intimate partner violence before, during, and/or after pregnancy was estimated to be 39.9% [95% CI 36.9–44.5]. About 18% of women reported continuous abuse over the perinatal period. Postpartum women who live in rural areas [adjusted odds ratio (AOR) = 2.46; 95% CI 1.21–5.01], or in neighborhoods with high IPV favoring norms [AOR = 1.49; 95%CI 1.01–2.20], high female literacy [AOR = 2.84; 95%CI 1.62–5.01], high female autonomy [AOR = 2.06; 95%CI 1.36–3.12], or in neighborhoods with lower wealth status [AOR = 1.74; 95%CI 1.14–2.66] were more likely to encounter PIPV. The complex patterns of interplaying factors operating at different levels could put pregnant or postpartum women at higher risk of IPV victimization. Therefore, policies that prioritize the improvement of contextual factors, particularly norms toward IPV and women’s empowerment are likely to be the most effective interventions.

## Introduction

Intimate partner violence (IPV) is a serious public health and human rights issue that affects individuals and families from all backgrounds^1,2^. This gross human rights violation involves physical violence, sexual violence, stalking, and/or psychological aggression to those in a close relationship^1^. Violence of any kind is unacceptable, but it is magnified when victims are pregnant or postpartum because of its detrimental effects on the mother, fetus, and newborns^3^. Perinatal IPV (PIPV) refers to violence committed by a partner before, during, and/or after pregnancy^4,5^. Ballard and colleagues identified four patterns of PIPV including violence begins (starts at pregnancy), violence continues (before and during pregnancy), violence ceases (before but not during pregnancy), and no violence (no violence at any stage)^6^. Although pregnancy, childbirth, and early parenthood are a joyful time for family; it can also be potentially stressful time due to significant changes in physical, psychological, social, and economic needs^4^. This unique period is linked to higher demands on individual capacities, couple relationships, and household economic resources, as well as reduction in a leisure time and opportunities to socialize, which may have a negative impact on emotional wellbeing^7^. When coping with such a stressful situation becomes difficult, the risk of psychological and physical aggression increases^8^. IPV victimization around pregnancy is very critical^9^ and can lead to adverse maternal and neonatal outcomes, such as hypertension, gestational diabetes, placental problems, infections, and mood disorders. Poor neonatal outcomes include preterm birth, small for gestational age, and low birth weight^10–12^.

In Ethiopia, nearly half of women experience at least one form of IPV in their lifetime^13^. According to the World Health Organization (WHO) report, the country ranks first in the world in terms of reporting violence against women^14^. Besides, one in the every three women fails to disclose partner abuse^15^. Unfortunately, limited number of studies has been conducted on the extent and continuity of IPV over perinatal period^4^. Presumably, understanding and identifying risk and protective factors is an important step for developing, implementing, and evaluating prevention and intervention strategies^16,17^. According to the socio-ecological model, studying contextual factors may help in better targeting interventions more appropriately for IPV victims and perpetrators^18^. Despite the fact that the nature of violence varies by community, there have been few studies on the community-level influences of perinatal violence^19^. Also, the existing evidences were based on single-factor theories, used no robust statistical analysis, and/or gave less attention to the roles of contextual factors that trigger or protect PIPV. Moreover, previous studies^20–22^ only measured violence during pregnancy and none has addressed the continuous nature of IPV across three mutually exclusive perinatal periods (before, during, and after pregnancy). Therefore, this study aimed to determine the prevalence of PIPV and to identify individual- and community-level factors associated with PIPV among postpartum population in the Wolaita zone, South Ethiopia.

## Methods and materials

### Study design and setting

This community-based cross-sectional study was conducted in the Wolaita zone of Ethiopia's South Nations, Nationalities, and People's Regions between October 2019 and January 2020. Administratively, the Zone is divided into sixteen rural districts (*‘Woredas’*) and six town administrations. It is one of the most densely populated areas in the region, with an estimated population of 2.5 million people. The number of women of reproductive age is estimated to be 582,500. The estimated number of postpartum populations among these women is 86,500.

### Source and study population with eligibility criteria

All postpartum women living in the Wolaita zone during the study period were considered the source population. The study population consisted of all the postpartum women in the zone’s randomly selected districts and towns. The study sample’s inclusion criteria were women of reproductive age who had lived with their current husband for at least 1 year, were within 6 weeks postpartum, had a permanent address, and had a current healthy infant. As this study was part of the prospective follow-up study designed to examine the interplay between self-reported PIPV and postpartum modern contraception, women who had not desired to become pregnant for 1 year were included in this study. The postpartum women who were not in a marriage, who had no intention of limiting or spacing births in the year following the survey, had a hysterectomy, or their husbands had a vasectomy, had a history of stillbirths and fetal deaths were excluded from the study.

### Sample size determination

The sample size was calculated using Epi-Info Version 7.0 by considering the single population proportion formula based on the following assumptions. The outcome variable was self-reported PIPV. As no similar study was conducted in the country to be used to determine the sample size, analysis from other developing countries was used. The proportion of postpartum women who reported PIPV in Nigeria was found to be 43.8% (p = 0.438)^23^. A 95% of confidence interval, a 4% margin of error, and a design effect of 2 were all considered. Finally, 10% was added for non-responses and miss to follow-up. The final sample was 1301. Considering factors associated with self-reported PIPV for double population proportion formula, decision-making power on household issues was found to be a strong factor in previous literatures^24^. The proportion of reported IPV among married women whose household issues are decided by husband only was found to be 68.6%, while proportion of IPV among women whose household issues are decided by jointly was assumed to be 53.6% by considering 15% risk difference, 95% CI, 80% power with a ratio of 1:4 (r = 4) and design effect of 2. Finally, 10% added for non-responses and the final sample size become 1236. However, this study was part of the prospective follow-up study designed to investigate the interplay between self-reported PIPV and postpartum family planning. The study had four specific objectives, and the alternative sample size for each objective was determined using both the double and single population proportion formulas. Of these alternative sample sizes, the maximum sample size (1320) was taken for all objectives considering the following assumptions: 95% CI, 4% margin of error, 80% power, proportion of postpartum modern contraceptive use (49%)^25^, design effect of 2, and 10% non-response rate. However, 1342 postpartum women who met inclusion criteria were approached at the time of the data collection to increase the power of the study.

### Sampling procedure

A multistage-clustered sampling technique was used to identify study participants. First, seven out of twenty-two districts in the zone (four rural districts and three town administrations) were selected using a simple random sampling method. These districts and towns were further clustered by 'Kebles,' Ethiopia's lowest administrative unit, and stratified into rural and urban Kebles. Second, thirty-eight Kebles (twenty-two rural and sixteen urban) Kebles were chosen randomly considering the number Kebles in each district. Then, sample size was allocated for each Keble using probability proportional to the size and the expected number of postpartum women per Keble. The lists of deliveries that took place within 6 weeks before the survey were refined and reconciled by data collectors from family folder of health extension workers (HEWs). In the case of households with more than one eligible woman, only one woman per household was chosen randomly. Finally, 1342 eligible women who met the inclusion criteria were sampled.

### Study variables and measurements

Data collection tool used can be found in the supplementary file (see Supplementary Table S1 online). *Dependent variables:* The outcome of the interest was self-reported perinatal partner violence. It was measured using section seven of the WHO standardized questionnaire^14^. A woman who reported at least one act of perinatal psychological, physical and sexual partner violence was coded as “1” for experiencing reported PIPV, and otherwise “0” (Cronbach’s α = 0.86). *Independent variables:* VAW integrated ecological framework^26,27^, followed as a guide to several factors associated with violence operating at different levels. The individual-level factors were specific to women, husbands, and relationship characteristics. The women-level factors included were women’s age at childbirth and marriage, education, employment status, religion, number of children, history of receiving bride-price at the wedding, women’s exposure to inter-parental violence, and attitudes that justify wife-beating. The partner-level factors examined were education, employment status, sex preferences, and alcohol/ substance abuse. The relationship-level factors considered for analysis were women’s participation in household decision-making, asset ownership, sex of index child, the couple’s age, and their income difference.

Women’s norms and attitudes towards IPV and a man’s control over his wife’s behaviors and activities were measured using sections six and seven of the WHO multi-country study on women’s health and domestic violence questionnaire^14,28^. Participants’ decision-making autonomy in household issues was measured^28^ by asking whether women participated in personal health care, daily household purchases, major household purchases, visits family or relatives, husband’s and her income (Cronbach’s α = 0.76). Community-level variables include women’s residency, classified as urban or rural based on the Ethiopian Central Statistical Authority descriptions of respondent’s location^29^. Other community-level variables were constructed by aggregating individual-level characteristics. The aggregates for clusters were computed using means (for normally distributed) or median (not normally distributed characteristics) in the woman’s cluster of residence. Finally, high-level variables were re-categorized into lower and higher categories.

### Data collection procedure

Data were collected using a pretested, interviewer-administered questionnaire adapted from other literature, including WHO and demographic and health survey (DHS) standard tools. The questionnaire was prepared in English, then translated to Amharic, and used to collect the data after back translation to English to check its consistency. According to WHO ethical and safety recommendations for research on domestic VAW^30^, training comprised of study’s aim and implementation, the basics of VAW, composition of questionnaire and interviewing techniques was prepared. Thirty-eight data collectors (married, female, diploma holders, bilingual) with eight supervisors (B.Sc. in Public Health) were recruited, trained, and deployed after receiving 2 days of intensive training. The training was given separately in each district for administrative purposes. All interviews were conducted in a private environment based on participants’ preferences. If the interview was interrupted by any person, the conversation about violence was changed to a questionnaire related to women’s health issues. At the end of the interview, participants’ district, Keble, village or got, name of women’s health development army (WHDA), head of WHDA, house and phone numbers, and name of the head of the household were recorded for relocating and arranging the study participants for a follow-up interview.

### Data management and analysis

Data were coded, entered into Epidata version 3.1, and exported to the SPSS for Windows 25 for descriptive analyses. The wealth status of participants was computed using principal component analysis (PCA). The hierarchical data with 1320 postpartum women nested in 38 clusters (Kebles) were constructed. The study participants within each cluster ranged from 20 to 43. Multilevel logistic regression models were used to determine associations between PIPV and individual- and community-level factors using STATA version 14. This model was preferred to avoid the clustering effects of factors operating at different levels on the outcome variable and violate the assumption of independence in standard logistic regression^31^. All significant variables at the p-value < 0.05 in bivariate analysis were considered candidates for multivariate analysis. Four Models were constructed in multivariate analysis.

The measures of association (fixed-effect) were shown as odds ratios at a 95% CI. Statistical significance was determined using a p-value < 0.05. In addition, to estimate the extent of variation (random effects) across communities, the models also include ICC, MOR, and PCV. ICC measures the proportion of the total heterogeneity that was attributable to the community level. It represents the ratio of the between-cluster variance to total variance^32,33^. However, MOR presents the cluster variance in the odds ratio scale. The MOR is the median value of the odds ratio between the area at the highest risk and the location at the lowest risk^34^. The PCV was also computed for each model concerning the unconditional model to present the power of the individual- and community-level factors in the models in explaining women’s experience of IPV^35,36^. Multicollinearity between the independent variables was checked using variance inflation factors (VIF). VIF value > 10 indicates that the presence of collinearity. Wherever multicollinearity existed, one of them was dropped from the model in turn. Akaike’s Information Criterion (AIC) was used for model selection, and the model with the lowest AIC value was considered the best-fitted model and used for description of the data^37^.

### Ethical considerations

The study was reviewed and approved by the Institutional Review Board of the College of Health Sciences, Addis Ababa University, with a protocol number of 006/19/SPH. The study followed and conducted with full respect of basic ethical principles of Helsinki declaration for medical research involving in human subjects^38^. All the study participants were briefed about the aim and procedures of the research and their right to abstain or withdraw from the study at any time. The informed consent was obtained from each participant separately. The confidentiality of the collected data was maintained by locking in the file cabinet. All study information was kept secured and confidential with the first author. After the interview, participants were allowed to visit a psychiatric nurse if they experienced any psychological discomfort.

## Results

### Basic background characteristics of currently married postpartum women in Wolaita Zone

Of the 1342 eligible women, 1292 (96.27%) participated in this study. The majority of participants, 57.1% were 25–34 years old with a mean age of 28.8 ± 5.6 years. Approximately 36% of the participants had never attended formal education, while 41% of their husbands had completed secondary or higher education. About 85% of the participants were unemployed and 35% of their husbands were in paid jobs. Approximately 18% of postpartum women witnessed inter-parental violence during childhood, and more than half, 57% had IPV favoring norms. About 37% of the postpartum women exposed to partner violence before index pregnancy. Regarding community-level characteristics, a large proportion of participants were living in a community with rural residence (56.3%), high early marriage (52.3%), high female literacy (55.7%), high IPV favoring norms (53.2%), high women’s decision-making autonomy (54.3%), and middle wealth status (34.0%) (Table 1).

**Table 1:** Individual-and community-level characteristics of currently married postpartum women in Wolaita zone, Southern Ethiopia, 2020.

Characteristics	Category	Frequency (N)	Percent (%)
**Woman-level characteristics**			
Maternal age (years)	≤ 24	295	22.8
	25–34	738	57.1
	35–49	259	20.1
Maternal age at marriage (in years)	< 18 years	399	30.9
	≥ 18 years	893	69.1
Religion	Orthodox christian	319	24.7
	Protestant christian	915	70.8
	Others*	58	4.5
Maternal education	No formal education	462	35.8
	Primary	401	31.0
	Secondary+	429	33.2
Maternal employment status	Not employed (non-salaried)	1099	85.1
	Employed	193	14.9
Number of living children	1–2	533	41.3
	3–4	465	36.0
	≥ 5	294	22.8
Sex of index child	Male	659	51.0
	Female	633	49.0
Received bridal price	No	555	42.9
	Yes	737	57.1
Justify intra-parental violence	No	1050	81.3
	Yes	242	18.7
Violence before the index pregnancy	No	810	62.7
	Yes	482	37.3
Justify wife beating norms	No	545	42.2
	Yes	747	57.8
Household wealth status	Poor	299	23.1
	Middle	673	52.1
	Rich	320	24.8
**Husband-level characteristics**			
Husband occupation	Non-employed	836	64.7
	Employed	456	35.3
Husband education	No education	388	30.0
	Primary	369	28.6
	Secondary+	535	41.4
Husband alcoholism	No	894	69.2
	Yes	398	30.8
Husband substance abuse	No	1116	86.4
	Yes	176	13.6
Intention for index pregnancy	Wanted pregnancy	1090	84.4
	Wanted delay	166	12.8
	Never minded it	36	2.8
Sex preferences of the index child	Male	586	45.4
	Female	229	17.7
	Never mind	477	36.9
Controlling behavior	No	611	47.3
	Yes	681	52.7
**Relationship-level factors**			
Years couple lived together	1–5 years	403	31.2
	6–10 years	500	38.7
	≥ 11 years	389	30.1
Decision-making autonomy	No	724	56.0
	Yes	568	44.0
Asset ownership (n = 764)	No	506	66.2
	Yes	258	33.8
Age difference	Younger than husband	1058	81.9
	The same in age	209	16.2
	Older than husband	25	1.9
Income difference	No income	810	62.7
	Earns less than	330	25.5
	Earns the same	62	4.8
	Earns more than	90	7.0
**Community-level factors**			
Place of residence	Urban	565	43.7
	Rural	727	56.3
Early marriage	High	682	52.8
	Low	610	47.2
Community-level women literacy	Low	572	44.3
	High	720	55.7
Community norms favoring IPV	Low	605	46.8
	High	687	53.2
Women’s decision-making autonomy	Low	590	45.7
	High	702	54.3
Wealth status	Poor	416	32.2
	Middle	439	34.0
	Rich	437	33.8

*=Others: Catholics, Muslim, Jehovah witness.

### Prevalence of self-reported perinatal partner violence against postpartum women in Wolaita zone, Southern Ethiopia (n = 1292)

**Figure 1:**
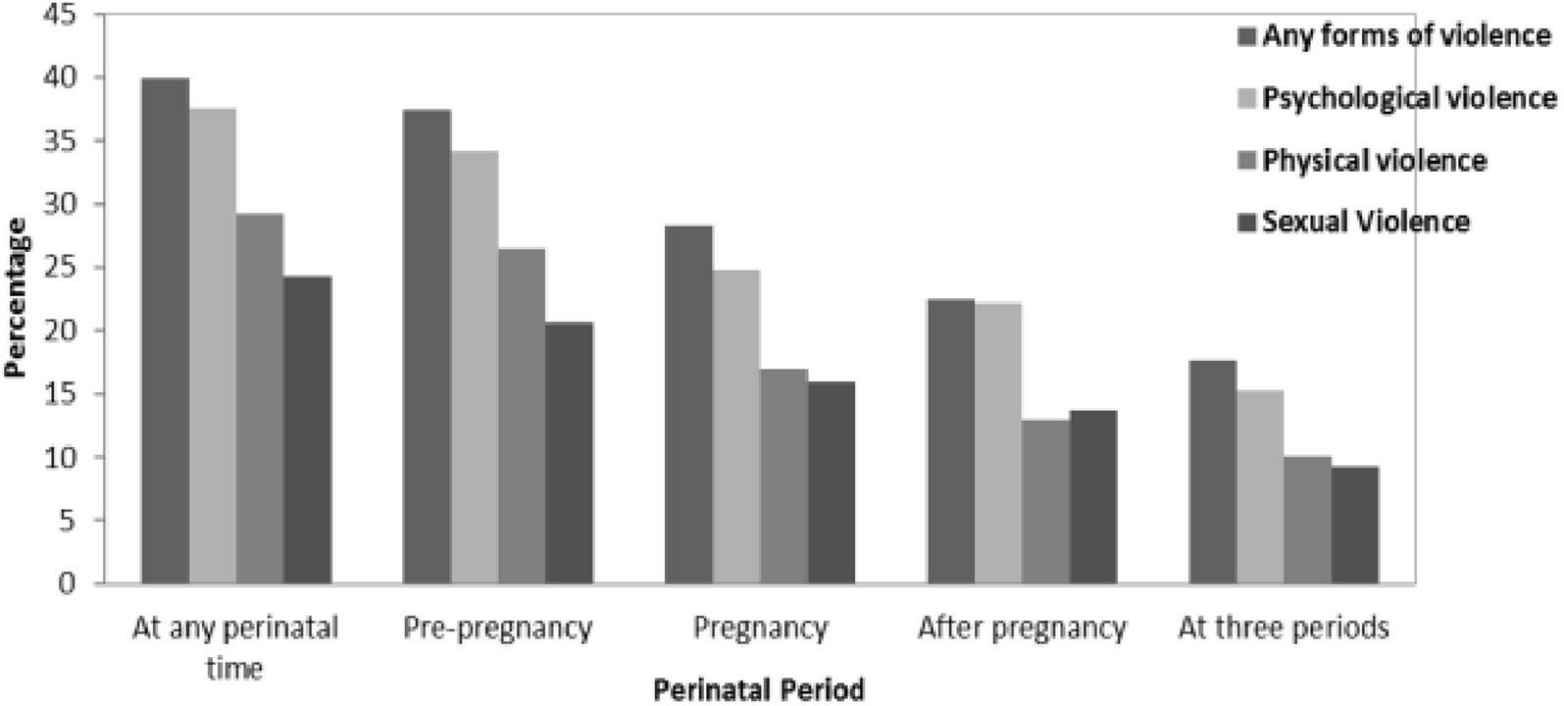
Patterns of self-reported perinatal partner violence according to the period of occurrence and its forms.

The overall prevalence of self-reported IPV over the perinatal period was 40%, where the most common type was psychological violence (37.6%) followed by physical violence (29.3%). The pattern of partner violence was changed over time. The overall prevalence of self-reported IPV before pregnancy was 37.4%, where psychological violence (34.2%) was high prevalent and sexual violence (20.7%) was low prevalent. The violence during pregnancy was 28.3%, where psychological violence (24.8%) was high, but comparable figures for physical (17.0%) and sexual violence (16.0%). Overall prevalence of violence in the postpartum period was 22.4% where psychological violence (22.2%) was high prevalent and comparative figures for physical violence (13%) and sexual violence (13.7%) (Fig. 1).

### Continuities in self-reported perinatal intimate partner violence in Wolaita Zone (n = 1292)

**Figure 2:**
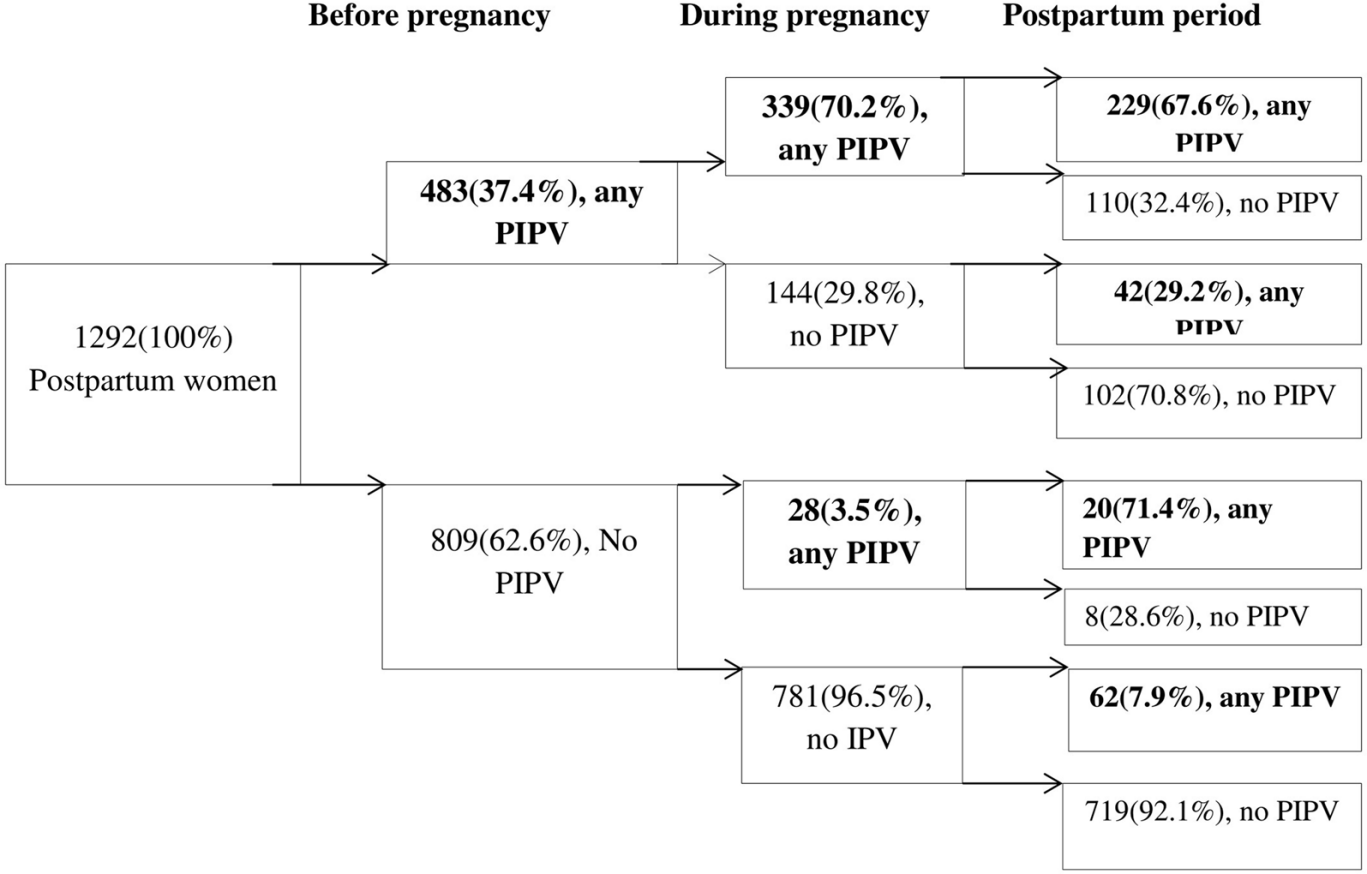
Continuities in perinatal intimate partner violence in Wolaita zone, Southern Ethiopia, 2020.

The continuity of self-reported PIPV was observed during the perinatal period (before, during, and after pregnancy). The continuity of the perinatal violence was calculated considering the reference point of the numbers of postpartum women “with” and “without” any PIPV during the preceding perinatal period. Out of 483 women who reported IPV within a year before pregnancy, about 70% of them experienced violence during their pregnancy (χ^2^ = 76.89, p ≤ 0.001). Of 367 postpartum women who experienced IPV during pregnancy, about 68% continuously reported after childbirth (χ^2^ = 35.16, p ≤ 0.001). Of the women who reported abuse before pregnancy, about 56% had experienced PIPV following childbirth (χ^2^ = 152.00, p ≤ 0.001). Of 809 postpartum women who were not abused before pregnancy, approximately 97% of them never experienced it during their pregnancy. Among those who experienced PIPV before pregnancy, approximately 70% encountered recurrent abuse during their pregnancy. Of those abused both before and during pregnancy, about 67% of them were also encountered continuous abuse following childbirth. Approximately 18% of the postpartum women experienced violence continuously over the entire perinatal period (Fig. 2).

### Community-level variance and model comparison of multilevel logistic regression by factors associated with perinatal violence among postpartum women in Wolaita Zone

#### Random effect results

The heterogeneity in PIPV experience between communities was measured using deviance, ICC, PCV, and MOR. The null model was fitted to verify suitability of multilevel analysis. ICC found to be 0.113, indicating that 11.3% of the total variability in experiencing PIPV was attributable to between cluster variations. The likelihood ratio test was strongly significant (p < 0.001) which favors the presence of clustering effect. Moreover, PCV indicated that individual-and community-level factors explained the 74% of the variation in experiencing PIPV across communities. Furthermore, MOR revealed the unexplained community variation in experiencing PIPV reduced from 2.12 (null model) to 1.47 (full model). This shows that if we randomly pick two individuals from two different communities, women in the community with a higher risk of PIPV had 2.1 times higher odds of experiencing PIPV compared with postpartum women in the community with a lower risk of PIPV (Table 2).

**Table 2:** The random-effects model and model comparison.

Random effects	Null model	Full model
Community-level variance	0.42	0.11
ICC (%)	11.3	3.2
PCV (%)	Reference	73.8%
Median odds ratio (MOR)	2.12	1.47
Model fitness statistics (AIC)	1733.624	1120.424
Model fitness statistics (BIC)	1743.952	1311.49
Log likelihood	-864.8122	–523.2121

#### Fixed effect results

In the full model, the effects of community-level variables largely emerged, but the association between self-reported PIPV and individual-level factors generally remained similar. Women from rural areas were 2.46 times more likely to encounter PIPV than their urban counterparts. Women from the community with high women literacy levels were 2.84 times more likely to experience PIPV compared to women from community with low literacy levels. The odds of violence were increased by 51% among women who lived in the community with high IPV favoring norms compared to those who lived in a community with low IPV favoring norms. The likelihood of PIPV among women from the community with high women’s autonomy was 2.06 times higher than women from the community with low women’s autonomy. However, odds of PIPV among women who participated in the decision-making process regarding household issues were decreased by 65% compared to those who did not. Women from the community with middle wealth status were 1.74 times more likely to experience PIPV than those with the richest wealth status. Postpartum women who attended no formal and primary education were 2.22 times and 1.60 times higher likelihood of experiencing PIPV than women who attended secondary or higher education, respectively. The odds of encountering partner abuse among women whose husbands attended primary and secondary and higher education were decreased by 49% and 39% compared to those whose husbands never attended formal education, respectively. Among postpartum women who reported IPV favoring attitude, the odds of PIPV were 3.35 times higher than women who did not justify wife-beating attitude, and the odds were 2.16 times higher among those who witnessed inter-parental violence during childhood than among those who had not. Women with alcoholic and wife controlling husbands were 1.71 times and 8.38 times more likely to experience PIPV than those who did not report such behaviors (Table 3).

**Table 3:** Multilevel logistic regression models for individual-, relationship-, and community-level factors associated with self-reported perinatal partner violence in Wolaita Zone (n = 1292).

Characteristics	Category	Exposure to perinatal IPV		Model II	Model III	Full model
		No	Yes	AOR (95%CI)	AOR (95% CI)	AOR (95% CI)
		Num. (%)	Num. (%)			
**Community-level factors**						
Place of residence	Urban	367 (65.0)	198 (35.0)	na	ref	ref
	Rural	410 (56.4)	317 (43.6)	na	2.22***	2.46*
Early marriage	High	346 (56.7)	264 (43.3)	na	ref	ref
	Low	431 (63.2)	251 (36.8)	na	1.08	1.22
Community norm favors IPV	Low	386 (56.2)	301 (43.8)	na	ref	ref
	High	391 (64.6)	214 (35.4)	na	0.92	1.49*
Community-level women literacy	Low	355 (62.1)	217 (37.9)	na	ref	ref
	High	422 (58.6)	298 (41.4)	na	1.86***	2.84***
Community-level women’s autonomy	Low	342 (58.0)	248 (42.0)	na	ref	ref
	High	432 (62.0)	267 (38.0)	na	1.09	2.06***
Community-level wealth Status	Poor	259 (62.3)	157 (37.7)	na	1.01	1.33
	Middle	240 (54.7)	199 (45.3)	na	1.26	1.74*
	Rich	278 (63.6)	159 (36.4)	na	ref	ref
**Woman-level factors**						
Woman’s age in years	≤ 24	134 (45.4)	161 (54.6)	2.04	na	2.07
	25–34	460 (62.3)	278 (37.7)	1.06	na	1.10
	35–49	183 (70.7)	76 (29.3)	ref	na	ref
Maternal age at marriage	< 18 years	193 (48.4)	206 (51.6)	1.15	na	1.20
	≥ 18 years	584 (65.4)	309 (34.6)	ref	na	ref
Maternal education	No formal	201 (43.5)	261 (56.5)	2.23***	na	2.22***
	Primary	251 (62.6)	150 (37.4)	1.61*	na	1.61*
	Secondary+	325 (75.8)	104 (24.2)	ref	na	ref
Employment status	Not employed	629 (57.2)	470 (42.8)	0.68	na	0.72
	Employed	148 (76.7)	45 (23.3)	ref	na	ref
No. of living children	1–2	309 (58.0)	224 (42.0)	ref	na	ref
	3–4	269 (57.8)	196 (42.2)	1.00	na	1.01
	≥ 5	199 (67.7)	93 (32.3)	1.12	na	1.14
Sex of index child	Male	434 (65.9)	225 (34.1)	ref	na	ref
	Female	343 (54.2)	290 (45.8)	1.29	na	1.22
Exposure to family violence	No	700 (66.7)	350 (33.3)	ref	na	ref
	Yes	77 (31.8)	165 (68.2)	2.18***	na	2.16***
Justify wife beating	No	450 (82.6)	95 (17.4)	ref	na	ref
	Yes	327 (43.8)	420 (56.2)	3.16***	na	3.35***
Wealth status	Poor	163 (54.5)	136 (45.5)	1.12	na	1.06
	Middle	406 (60.3)	267 (39.7)	0.82	na	0.84
	Rich	208 (65.0)	111 (35.0)	ref	na	ref
**Partner-level factors**						
Husband occupation	Non-employed	483 (57.8)	353 (42.2)	0.91	na	0.92
	Employed	294 (64.5)	162 (35.5)	ref	na	ref
Husband education	No education	174 (44.8)	214 (55.2)	ref	na	ref
	Primary	231 (62.6)	138 (37.4)	0.53**	na	0.51**
	Secondary+	372 (69.5)	163 (30.5)	0.63	na	0.61*
Husband alcoholism	No	612 (68.5)	282 (31.5)	ref	na	ref
	Yes	165 (41.5)	233 (58.5)	1.73***	na	1.71**
Husband substance abuse	No	712 (63.8)	404 (36.2)	ref	na	ref
	Yes	65 (36.9)	111 (63.1)	1.14	na	1.13
Intention of index pregnancy	Intended	687 (63.0)	403 (37.0)	ref	na	ref
	Not intended	75 (45.2)	91 (54.8)	3.56***	na	3.17***
	Never minded	15 (41.7)	21 (58.3)	3.23*	na	2.98*
Husband’s sex preferences	Male	311 (53.1)	275 (46.9)	0.99	na	0.90
	Female	162 (70.7)	67 (29.3)	0.63	na	0.62
	Never minded	304 (63.7)	173 (36.3)	ref	na	ref
Controlling behavior	No	526 (86.1)	85 (13.9)	ref	na	ref
	Yes	251 (36.9)	430 (63.1)	8.66***	na	8.38***
**Relationship-level factors**						
Years couple lived together	1–5 years	232 (57.6)	171 (42.4)	1.25	na	1.28
	6–10 years	274 (54.8)	226 (45.2)	2.12***	na	2.15***
	≥ 11 years	271 (69.7)	118 (30.3)	ref	na	ref
Decision-making autonomy	No	324 (44.8)	400 (55.2)	ref	na	ref
	Yes	453 (79.8)	115 (20.2)	0.37***	na	0.35***
Age difference	Younger than	633 (59.8)	425 (40.2)	0.42	na	0.39
	The same	133 (63.6)	76 (36.4)	0.31*	na	0.28*
	Older than	11 (44.0)	14 (56.0)	ref	na	ref

Statistically significant at *p-value < 0.05, **p-vale ≤ 0.01, ***p-value < 0.001, ref = reference group, na = not applicable.

## Discussion

The current study shows that about 40% (95% CI 36.9–44.6) of women had experienced intimate partner violence before, during, and/or after pregnancy. This finding is consistent with clinical studies conducted in Southern Nigeria (43.8%) and Tanzania(43%)^23,39^, but lower than study conducted in Brazil (47.4%) and Iran (64.7%)^40,41^. Despite these comparative figures from clinical settings which yield high prevalence rates, this community-based finding confirms that a significant proportion of postpartum women are at risk for PIPV. The prevalence of PIPV was decreased over perinatal periods, the highest in the year before pregnancy (37.4%) and lowest after childbirth (one and half months) (22.4%), which accords with studies conducted elsewhere^41–43^. This lowest incidence of abuse after childbirth can be attributed with the study period variability of the postpartum period and cultural celebrations and presence of extended family following successful childbirth. Another explanation could be linked to fear of vulnerability that perinatal women in an abusive relationship may try to protect against being harmed by using techniques such as hiding and avoidance. In this study, low prevalence of physical and sexual violence was observed over the perinatal period. This finding corroborates with other studies that have identified low incidence of physical and sexual violence during the perinatal period^39,42,44^. The possible justifications could be the husband’s fear of the social stigma associated with wife battering or decreased sexual demands in this formative period. Most importantly, caution should be taken when interpreting the reduction of abuse over perinatal periods. The evidence indicates that existing abuse escalates in frequency and severity in the perinatal periods^43,45,46^. Our study found that over two-thirds of women who reported IPV before pregnancy also experienced continuous abuse during and after pregnancy. This result confirms the fact that once abuse has initiated, it will continue during the transition to parenthood.

In this study, being a rural resident was associated with high PIPV encountering. This finding corroborating with prior studies^20,47^. In contrast, urban residency was also a trigger for PIPV^48^. Again, paradoxically to other studies^49–51^, being in urban places was found to be a protective factor against PIPV. This might be because living in urban areas may offer women more opportunities to access media outlets, economic resources, institutional supports, and new information, which can help them cope with violence more effectively. Consistent with the social causation theory^52^, the current study shows a reciprocal relationship between women’s education status and PIPV. Increasing women’s education reduces any form of the recent and long-term probabilities of IPV, which is supported by past research conducted in Pakistan, Belgium, and the USA^53–55^. In contrast to a study done in India^56^, neighborhoods with high women’s literacy were linked with an increased risk of PIPV. This might be due to interaction with traditional gender ideology in a patriarchal society that expects women to be submissive in all spheres of marital relationships, which may not work for more educated women and could lead to violence. In this study, women from the community with low wealth terciles were also at increased risk of PIPV as evidenced by studies conducted elsewhere^57,58^. This result may imply that any violence prevention strategies should prioritize women living in neighborhoods with the lowest wealth terciles. At individual level, postpartum women’s decision-making autonomy in household issues was found to be protective for PIPV in the current study. Conversely, women concentrated in the community with high women decision-making autonomy have a high probability of encountering PIPV. This suggests that as women gain autonomy, they struggle for reproductive autonomy, including fertility control, which can lead to PIPV victimization in traditional societies where men hold primary decision-making power in marriage, as evidenced by other studies^59–61^. Also, the result is consistent with a cohort study conducted in Nepal, which found that the risk of contracting IPV was higher in women who became pregnant and gave birth than in those who did not^62^. This implies that ensuring women’s decision-making autonomy requires addressing IPV and related constraints.

In agreement with social learning theory, postpartum women’s witness to inter-parental abuse during childhood was linked with increased PIPV victimization. Similarly, a study conducted in Brazil reveals that witnessing or being a victim of family violence was associated with being perpetrators or victims of PIPV when becoming adults^63^. The possible reason may be exposed to family violence can cause many women to become determined not to tolerate violence in their marriage. In the current study, women who endorsed wife-beating norms and living in the community with high IPV favoring norms were at increased risk of PIPV. This implies harmful traditional models play a vital role in sustainability of violence and need to be cured through social norms intervention. In a replication of previous studies in Malaysia, Brazil, and the USA^64–68^, the husband’s alcohol misuse and partner controlling behavior were associated with high PIPV victimization. In line with a study conducted in the USA^8^, unintended index pregnancy was also a triggering factor for PIPV. The association could be explained in different ways. An abusive partner could limit the woman’s ability to control her own fertility or because the woman in a violent relationship may neglect to take care of their fertility control needs, which could lead to unintended pregnancies. Inconsistent with a study conducted in Nepal^44^, a short duration of marriage was a risk for PIPV. The possible reason could be a lack of awareness on coping with stress and changes during the childbearing period for couples in the short duration of cohabitation. Similarly, a couple’s age difference predicts PIPV. Being the same age as a husband protects perinatal abuse as being older. This finding implies interventions that consider and reduce women’s high age disparity in the community are needed to reduce the vulnerability of women to PIPV. Contrary to other studies^69,70^, infant gender and son preferences were not predicted PIPV encountering. This finding also contradicts the researchers’ early results from a qualitative study^71^. This requires further investigations.

This study has significant limitations while using this research finding. First, we could not establish a causal relationship due to the cross-sectional nature of the study design. Second, there are limited community-based cluster-level studies to compare this finding, which demarcated us to compare results from clinical studies to the population-based studies. Third, as researching violence against women suffers from under-reporting, this study was not free from this problem despite all data supervisors and enumerators being well-trained. Fourth, exclusion of women: unmarried, in the extended postpartum periods, and had a stillbirth or neonatal death was another limitation because violence rates might be high in these groups. Despite these limitations, this study has some important implications. To the best of our knowledge, this is the first community-based study in the country that investigates the community-level variation of self-reported perinatal violence among postpartum women. Relatively, the study was focused on recent self-reported IPV and may indicate the assumption of less recall bias. Indeed, experts in this area suggest that a woman never forgets her husband’s action following pregnancy and childbirth, whether treating poorly or disrespecting her over the perinatal period will scar her for life. Thus, any abuse from their husband is recalled with great clarity.

## Conclusion

Our study found that about one-fifth (18%) of postpartum women are continuously subjected to partner violence over the perinatal period. A significant heterogeneity was observed between clusters in PIPV victimization. The complex patterns of interplaying factors operating at different levels could put pregnant or postpartum women at higher risk of perinatal abuse. Therefore, policies that prioritize the improvement of contextual factors, particularly norms toward IPV and women’s empowerment, are likely to be the most effective interventions with multidisciplinary and intersectoral collaborations. In addition, nationally appropriate guidelines, strategies, and programs should be prepared that prioritize and support perinatal women at risk of IPV. Further, future studies that investigate the role of social processes and norms that help IPV sustainability among perinatal women are also suggested.

## Supplementary Information

Supplementary Table S1.

## Data Availability

The data analyzed during the current study are available from the corresponding author upon reasonable request.
