# Supplementary material for: Multilevel analysis of factors associated with perinatal intimate partner violence among postpartum population in Southern Ethiopia
Source: Sci Rep. 2022 Nov 8;12:19013. doi: 10.1038/s41598-022-23645-4 (PMC9643427; doi:10.1038/s41598-022-23645-4)
Supplement: Supplementary file 1 — Supplementary Table S1. [file 41598_2022_23645_MOESM1_ESM.pdf]

Multilevel analysis of factors associated with perinatal intimate partner violence among postpartum population in Southern Ethiopia

Tafesse Lamaro Abota [1, 2*], Fikre Enqueselassie Gashe[2]☦, Negussie Deyessa[2]

[1] College of Medicine and Health Sciences, Mizan-Tepi University, Mizan-Aman, Southwest Ethiopia

[2]School of Public Health, College of Health Sciences, Addis Ababa University, Addis Ababa, Ethiopia.

* Corresponding Author's email address: lamaro.tafesse@yahoo.com

☦ Deceased

Supplementary Table S1: Data collection tool on postpartum women's health and life events in Wolaita zone, Southern Ethiopia

General background of survey participants and screening format to participate in the survey (Filled by data collectors before commencing face-to-face interview)				
**No.**	**Questions and Filters**	**Coding category**	**Address Name**	**Skip**
01	Participant located using Woreda	1 Yes 2. No	_______________	
02	Participant located using Kebles	1. Yes 2. No	_______________	
03	Participant located by Village/block/Got	1. Yes 2. No	_______________	
04	Participant located by household head	1. Yes 2. No	_______________	
05	Participant located by house number	1. Yes 2. No	_______________	
06	Participant located by phone address	1. Yes 2. No	_______________	
07	Code of Questionnaire_____________	Woreda_____Keble_____Women code________		
	**Name of data collector ____________________sig. _________ Date _______________**<br>**Name of data Supervisor:_________________ sig. _________Date _______________**<br>**Date of data interview_____________________ /___________/_____________**			

Section 1: Sociodemographic characteristics of the study participants in the Wolaita zone, Southern Ethiopia			
Sr.No	**Questions**	**Response Category**	**Skip patterns**
101	Where is your area of residence?	1. Rural 2. Urban	
102	How old were you at your last birthday?	Age in the completed years________	
103	What is your Ethnicity?	1. Wolaita<br>2. Gurage<br>3. Amhara<br>4. Dawuro<br>5. Others Specify_________	
104	What is your religion?	1. Orthodox Christian<br>2. Protestant Christian	

		3. Catholic<br>4. Muslim<br>5. Others specify___________	
105	Have you ever attended school?	1. Yes 0. No (illiterate)	→Q#107
106	If Yes to Q105, what is the highest level of school you attended?	_________Grade<br>1. Primary (1-8 grade)<br>2. Secondary(9-12 grade)<br>3. Technical/vocational (10+3 and above)<br>4. Higher(College and above)	
107	Had your mother ever attended school?	1. Yes 0.No 3. I don't know	
108	If Yes to Q107,what is the highest level of school your mother attended:	___________Grade<br>1. Primary (1-8 grade)<br>2. Secondary(9-12 grade)<br>3. Technical/vocational (10+3 and above)<br>4. Higher(College and above)	
109	What is your current occupation?	1. Housewife/farmer<br>2. Government employee<br>3. Private employee<br>4. NGO employee<br>5. Merchant<br>6. Student<br>7. Others specify _____________	
110	In addition to your house work, do you have any other work for which you are paid in cash or in kind?	1. Yes 0. No	If No,→Q #112
111	If Yes to Q110, on average how much birr is paid for you per month?	_______ (ET. Birr)	
112	Do you usually work throughout the year, or do you work seasonally, or only once in a while?	1. Throughout the Year<br>2. Seasonally/Part of the Year<br>3. Once in a while	
113	What age were you when you married?	__________years	
114	How long have you been married to your current husband?	__________years	
115	How much you are younger or older than your partner?	____________Years<br>1. Younger than spouse<br>2. The same age as spouse<br>3. Older than spouse	

116	Did you or your family receive price from the bride's or groom's family in kind or cash at the time of your marriage?	1. Yes 0. No 3. I don't Know	
117	How you level the cost of bridal price according to groom and his family economic status?	1. Little dowry<br>2. Some dowry<br>3. huge dowry<br>4. I didn't remember it	
118	What is current occupation of your husband?	1. Farmer<br>2. Government employee<br>3. private employee<br>4. NGO employee<br>5. Merchant<br>6. Student<br>7. Others specify ___________________	
119	What is approximate monthly income of your husband per month?	__________ (ET. Birr)	
120	How label your earnings when compared to your spouse?	1. Earns less than spouse<br>2. Earns same as spouse<br>3. Earn more than spouse<br>4. Woman has No earning	
121	Have your husband ever attended school?	1. Yes 0.No 3. I don't Know	If No & DK; skip to 224
122	If Yes to Q121, What is the highest level of school your attended?	____________Grade<br>1. Primary (1-8 grade)<br>2. Secondary(9-12)<br>3. Technical/vocational (10^{+3})<br>4. Higher(College and above)	
123	How label your educational status when compared to your spouse?	1. Less educated than spouse<br>2. The same education as spouse<br>3. More educated than spouse	
124	How many inhabitants residing in this house including you?	______________Inhabitants	
125	Does your household own the following?		
	Electricity?	1. Yes 0. No	
	Radio?	1. Yes 0. No	
	Television?	1. Yes 0. No	
	Computer	1. Yes 0. No	

	Non-mobile telephone functioning?	1. Yes 0. No	
	Refrigerator?	1. Yes 0. No	
	Table?	1. Yes 0. No	
	Chairs?	1. Yes 0. No	
	A bed with cotton/ sponge/ spring mattress?	1. Yes 0. No	
	An electric 'Mitad'?	1. Yes 0. No	
	A kerosene lamp/pressure lamp?	1. Yes 0. No	
126	Does any member of your household own the following?		
	Watch?	1. Yes 0. No	
	Mobile phone?	1. Yes 0. No	
	Bicycle?	1. Yes 0. No	
	Motor cycle?	1. Yes 0. No	
	Animal drawn cart?	1. Yes 0. No	
	A car or truck	1. Yes 0. No	
127	Does any member of this household own any agricultural land?	1. Yes 0. No	If No skip to Q 129
128	If Yes to Q126, how many hectares?	_____________Hectares	
129	Does this household own any livestock, herds, other farm animals, or poultry?	1. Yes 0 No	If No skip to Q#201
130	If yes to Q128, how many:	_______________	
	Cattle?	_______________	
	Milk cows or bulls	_______________	
	Horses, donkeys or Mules?	_______________	
	Goats?	________________	
	Sheep?	________________	
	Chickens?	________________	

Section-2: Obstetric and Reproductive history of study participants in Wolaita zone			
201	How many children do you currently have?	Male _____Female______ Total____	
202	How many pregnancies have you ever had?	__________	
203	Did any of these pregnancies ended in abortion (termination of pregnancy before 28 weeks of gestation)?	1.Yes 0.No	If No skip to Q#205
204	If Yes to Q 203, how many of them ended in abortion?	______times	
205	How many of your pregnancies were unintended?	_________	
206	What is the name of your index baby?<br>Sex of index baby?	Name:------------------------------------<br>Male……………………………..1<br>Female……………………………2	
207	In what month and year was your index baby born?<br>Age of index baby?	Date_____Month______Year________<br>Age ______in months	
208	Is your most recent pregnancy a planned one? (**Read the options**)	1.Wanted to become pregnant<br>2. Wanted to delay pregnancy<br>3.Unwanted pregnancy<br>4. I didn't thought about it<br>5.Don't know<br>6. Not agreed to answer	
209	If your answer is ''1'' for Q208, have you used anything or tried in any way to delay or avoid getting pregnant?	1. Yes 0. No 3 Don't know	
210	If Yes for Q209, which one of the following contraception was used to prevent latest pregnancy?	1. IUCD<br>2. Implants<br>3. Injectables<br>4. Pill<br>5. Male condom	

		6. Female condom<br>7. Emergency contraception<br>8. Traditional methods<br>9. Others specify______________________	
211	What was the most important reason you stopped using this method?	1. Wanted to become pregnant<br>2. Became pregnant while using<br>3. Side effects<br>4. Health concerns<br>5. Difficult to use<br>6. Infrequent sex/husband away<br>7. Husband/partner disapproved<br>8. Wanted more effective method<br>9. Lack of access/too far<br>10. Costs too much<br>11. Difficult to get pregnant/menopausal<br>12. Marital dissolution/separation<br>13. Missed appointment<br>14. Method not available<br>15. Other(Specify)______________________<br>16. Don't know	
212	Regarding your latest pregnancy, what was your husband's condition? (Read the options)	1.He wanted pregnancy<br>2. He wanted to delay pregnancy<br>3.He didn't want a child now<br>4. I didn't mind about it<br>5.I didn't know/remember<br>6. Not agreed to give an answer	
213	Regarding your index pregnancy; what was your husband's sex preferences?	1. Male<br>2. Female<br>3. He didn't mind it<br>4. I didn't know/remember<br>5. Not agreed to give an answer	
214	Where did you give your last delivery?	1. Hospital<br>2. Health center<br>3. Health post<br>4. Home<br>5. Other (specify) _____	
215	How much longer did you want to wait before you became pregnant for this birth (change into months)?	_________months	
216	Have you used any contraceptive methods since this birth?	1. Yes<br>0. No	If No; skip to 221

217	What was your first family planning methods adopted since this birth?<br>**(Multiple responses are possible)**	1. IUCD<br>2. Implants<br>3. Injectable<br>4. Pill<br>5. Male condom<br>6. Female condom<br>7. Emergency contraception<br>8. Traditional methods<br>9. Others specify____________________	
218	If your answer is ‘’Yes’’ for Q217; when you initiated first methods since this birth?	Date______ Month________Year_____	
219	If your answer is ‘’Yes’’ for Q217, up to what month and year have you been using (current methods) without stopping?	Date______ Month________Year_____	
220	If Yes for Q217,reasons for contraceptive adoption	1. Spacing 2. Limiting 3. Unknown	
221	If your answer is ‘’ No’’ for Q217, reasons for not adopting contraception?	1. Breast feeding<br>2. Postpartum abstinence<br>3. No resumption of menses<br>4. counselled by health professionals<br>5. Partner not wanting<br>6. Friends not supporting<br>7. others specify____________________	
22	Are you currently breastfeeding?	1. Yes 0. No 3. Don’t know	
223	How many times did you breastfeed last night between 6:00p.m. and 6:00a.m.?	Number of night time feedings_______________	
224	How many times did you breastfeed yesterday between 6:00a.m. and 6:00p.m.?	Number of day time feedings<br>__________________	
225	Average duration of breastfeeding in each episode	_______________in minutes!	
226	Has your menstrual period returned since this birth?	1. Yes 0. No 3. I don’t know	If No skip to Q#228
227	When your first menses have resumed since this birth?	Date______ Month________Year_____	
227B.	For how many months after the birth of index child, did you not have a period?	1. Months________/________<br>2. Don’t know	

228	From one menstrual period to the next, are there certain days when a woman is more likely to become pregnant?	1. Yes<br>0. No<br>3 I don't know	
229	If your answer is ''YES'' for Q 228,is this time just before her period begins, during her period, right after her period has ended, or halfway between two periods?	1. Just before her period begins<br>2. During her period<br>3. Right after her period has ended<br>4. Halfway between two periods<br>5. Other specify ________________<br>6. I don't know	
230	After the birth of a child, can a woman become pregnant before her menstrual period has resumed?	1. Yes<br>0. No<br>3 I don't know	
231	Have you had sexual intercourse since this birth?	1. Yes 0. No	
232	When was your first sexual intercourse since this birth**?(Read options)**	Date______ Month________Year_____<br><br>1.Before menses resumes<br>2.After menses resumes<br>3.Before contraceptive adoption<br>4.After contraceptive adoption	
232B.	For how many months after the birth of index child; did you not have sexual intercourse?	Months_______/________<br>Don't know	
***Reproductive double column calendar was used to measure contraceptive adoption and discontinuation**			
**Section 3: Household's decision making, asset ownerships and women spouses' characteristics**			
301	Who usually decides how to spend the money that you earn? You, your husband/partner, both, or someone else?	1. Yourself<br>2. Your husband<br>3. Jointly (you and your husband)<br>4. Someone else	
302	Who usually decides how your husband's earnings will be used: you, your husband, or you and your (husband/partner) jointly?	1. Alone<br>2. My husband<br>3. Jointly(you and your husband)<br>4. Someone else	
303	Who usually makes decisions about health care for yourself: you, your (husband/partner), you and your (husband/partner) jointly, or someone else?	1.Alone<br>2. My husband<br>3.Jointly(you and your husband)<br>4.Someone else	

304	Who usually makes decisions about making major household purchases?	1.Yourself<br>2.Husband<br>3.Jointly(you and your husband)<br>4.Someone else	
305	Who usually makes decisions about making daily household expenses?	1.Yourself<br>2.Husband<br>3.Jointly(you and your husband)<br>4.Someone else	
306	Who usually makes decisions to visit family or relative?	1.Yourself<br>2.Husband<br>3.Jointly(You and your husband)<br>4.Someone else	
307	Does your husband help you with household chores like looking after children, cooking, cleaning the house and doing other work around the house?	1.Yes<br>0. No	If No, skip to 309
308	Does he help you almost every day, at least once a week or rarely?	1. Almost every<br>2. At least once a week<br>3. Rarely	
309	Do you own this or any other house either alone or jointly with someone else?	1. Alone only<br>2. Jointly only<br>3. Both alone and jointly<br>4. Does not own	If Does not own, Skip to 312
310	Do you have a title deed for any house you own?	1. Yes<br>0. No<br>3 Don't know	If No and DK,skip to 312
311	Is your name on the title deed?	1. Yes<br>0 No<br>3 Don't know	
312	Do you own any agricultural or non-agricultural land either alone or jointly with someone else?	1. Alone only<br>2. Jointly only<br>3. Both alone and jointly<br>4. Does not own	
313	Is your name on the title deed?	1.Yes<br>0 No<br>3.Don't know	

314	Do you have a title deed for any land you own?	1. Yes<br>0 No<br>3 Don't know	
315	How you label alcohol (Areke,Teji,Tela,Beer,wine etc.) Consumption status of your husband?	1. Does not drink<br>2. Drinks/never gets drunk<br>3. Get drunk sometimes<br>4. Gets drunk very	
316	How your husbands chew chat?	1. Daily<br>2. 1 to 2 times in a week<br>3. 1 to 3 times in a month<br>4. Never chew chat<br>1I don't remember<br>5. I don't know	
317	Is your partner use tobacco?	1. Yes 0. No 3. Don't Know	
318	Did your husband engaged in any conflicts with anybody since you're engaged in this marriage?	1. Yes<br>0 No<br>3 Don't know	
319	If yes for Q318, how often did he engage in conflict in last 12 months?	1.Daily<br>2. 1 to 2 times<br>3. 3 to 5 times<br>4. More than 5 times<br>5. I don't remember<br>6. I don't know	
320	Did your current husband have any relationship with other women out of you?	1.Yes<br>2. No<br>3.It may be<br>4.I don't know<br>5. Not agreed to answer	
321	Did your husband born any children from other women since this marriage?	1.Yes<br>2. No<br>3.It may be<br>4.I don't know<br>5. Not agreed to answer	
322	Did you have an exposure as a child, whether your current/ex father had ever beaten your mother?	1.Yes<br>0. No<br>3.Don't know	
323	Do you think violence from your husband is normal?	1.Yes 0. No 3 Don't Know	
324	In your opinion, is a husband justified in hitting or beating his wife in the following situations:<br>a.) If she goes out without telling him?<br>b.) If she neglects the children?<br>c.) If she argues with him?	Yes No DK<br>a. Goes out 1 2 3	

	d.) If she burns the food?<br>e.) If she refuses to have sex with him?	b. Neglect children 1 2 3<br>c. Argues 1 2 3<br>d. Refuses sex 1 2 3<br>e. Burns food 1 2 3	
325	In your opinion, when a woman can refuse sex with her husband?	Yes No DK<br>a. Not wanted 1 2 3<br>b. He gets drunk 1 2 3<br>c. Sick/not in mood 1 2 3<br>d. Engage in conflict 1 2 3	

Session 4: Perinatal intimate partner violence among postpartum women in the Wolaita zone, Southern Ethiopia.

When two people marry or live together, they usually share both good and bad moments. I would now like to ask you some questions about your current and past relationships and how your husband / partner treat (treated) you. If anyone interrupts us; I will change the topic of conversation. I would again like to assure you that your answers will be kept secret, and that you do not have to answer any questions that you do not want to. May I continue?

401 . With your current husband; did you have any communication in following issues?

	Did you communicate your days with husband?	1. Yes 2. No 3. DK	
	Did your husband communicate his day with you?	1. Yes 2. No 3. DK	
	Did you share daily stressful events with your husband?	1. Yes 2. No 3. DK	
	Did your husband share daily stressful events with you?	1. Yes 2. No 3. DK	

402. How often times you have engage in conflict with your current husband?

Rarely……..1 Sometimes……2 Always…..3 I don’t know/remember ………4 Refused to answer….1

403	**I am now going to ask you about some situations that are true for many women. Thinking about your**

	**former/current husband, would you say it is generally true that he:**			
	A. Tries to keep you from seeing your friends?	1. Yes 2. No 3. DK		
	B. Tries to restrict contact with your family of birth?	1. Yes 2. No 3.DK		
	C. Insists on knowing where you are at all times?	1. Yes 2. No 3.DK		
	D. Ignores you and treats you in differently?	1. Yes 2. No 3.DK		
	E. Gets angry if you speak with another man?	1. Yes 2. No 3.DK		
	F. Is often suspicious that you are unfaithful?	1. Yes 2. No 3. DK		
	G. Expects you to ask his permission before seeking	1. Yes 2. No 3.DK		
404	The next questions are about things that happen to many women, and that your former/current partner, or any other partner may have done to you.	A)<br>**a)** Has this happened in the 12 months before index pregnancy?<br>***If ' Yes'→''b''***<br>***If ' No'→''B''***<br>**b)** Was it happened once, a few times or many times?	B)<br>**a)** Has this happened during index pregnancy?<br>***If ' Yes'→''b''***<br>***If ' No'→''C''***<br>**b)** Was it happened once, a few times or many times?	***C)***<br>**a)** Has this happened following index child birth?<br>***If ' Yes'→''b''***<br>***If ' No'→''405''***<br>**b)** Was it happened once, a few times or many times?
	Did you experience the following events from your former/current partners in three periods (before, during and after pregnancy)?	***1. Yes 2. No → B)***<br>***↓***<br>***1.Once 2.Few 3.Many***	***1. Yes 2. No → C)***<br>***↓***<br>***1.Once 2.Few 3.Many***	***1. Yes 2. No → 405)***<br>***↓***<br>***1.Once 2.Few 3.Many***
	A. Insulted you or made you feel bad about yourself?	1 2<br>1 2 3	1 2<br>1 2 3	1 2<br>1 2 3
	B. Belittled or humiliated you in front of other people?	1 2<br>1 2 3	1 2<br>1 2 3	1 2<br>1 2 3

	**C.** Done things to scare or intimidate you on purpose (e.g. by the way he looked at you, by yelling and smashing things)?	1 2<br>1 2 3	1 2<br>1 2 3	1 2<br>1 2 3
	D. Threatened to hurt you or someone you care about?	1 2<br>1 2 3	1 2<br>1 2 3	1 2<br>1 2 3
405	***Did you experience the following events from your former/current partners in three periods (before, during and after pregnancy)?***	A)<br>**a)** Has this happened in 12 months before latest pregnancy?<br>***If ' Yes'→''b''***<br>***If ' No'→''B''***<br>**b)** Was it happened once, a few times or many times?	B)<br>**a)** Has this happened during latest pregnancy?<br>***If ' Yes'→''b''***<br>***If ' No'→''C''***<br>**b)** Was it happened once, a few times or many times?	***C)***<br>**a)** Has this happened following index child birth?<br>***If ' Yes'→''b''***<br>***If ' No'→''406''***<br>**b)** Was it happened once, a few times or many times?
		1. Yes 2. No → B)<br>↓<br>***1.Once 2.Few 3.Many***	***1. Yes 2. No → C)***<br>↓<br>***1.Once 2.Few 3.Many***	***1. Yes 2. No → 406)***<br>↓<br>***1.Once 2.Few 3.Many***
	A. Slapped you or thrown something at you that could hurt you?	1 2<br>1 2 3	1 2<br>1 2 3	1 2<br>1 2 3
	B. Pushed you or shoved you or pulled your hair?	1 2<br>1 2 3	1 2<br>1 2 3	1 2<br>1 2 3
	C. Hit you with his fist or with something else that could hurt you?	1 2<br>1 2 3	1 2<br>1 2 3	1 2<br>1 2 3
	D. Kicked you, dragged you or beat you up?	1 2<br>1 2 3	1 2<br>1 2 3	1 2<br>1 2 3
	E. Choked or burnt you on purpose?	1 2<br>1 2 3	1 2<br>1 2 3	1 2<br>1 2 3

	F. Threatened to use or actually used a gun, knife or other weapon against you?	1 2<br>1 2 3	1 2<br>1 2 3	1 2<br>1 2 3
406	**Did you experience the following events from your current partners in three periods (before, during and after pregnancy)?**	A)<br>**a)** Has this happened in the 12 months before latest pregnancy?<br>***If ' Yes'→''b''***<br>***If ' No'→''B''***<br>**b)** Was it happened once, a few times or many times?	B)<br>**a)** Has this happened during latest pregnancy?<br>***If ' Yes'→''b''***<br>***If ' No'→''C''***<br>**b)** Was it happened once, a few times or many times?	C)<br>**a)** Has this happened following index child birth?<br>***If ' Yes'→''b''***<br>***If ' No'→''407''***<br>**b)** Was it happened once, a few times or many times?
		1. Yes 2. No → B)<br>↓<br>***1.Once 2.Few 3.Many***	***1. Yes 2. No → C)***<br>↓<br>***1.Once 2.Few3.Many***	***1. Yes 2. No → 407)***<br>↓<br>***1.Once 2.Few 3.Many***
	A. Did your former/current husband ever physically force you to have sexual intercourse when you did not want to?	1 2<br>1 2 3	1 2<br>1 2 3	1 2<br>1 2 3
	B. Did you ever have sexual intercourse you did not want to because you were afraid of what your partner or any other partner might do?	1 2<br>1 2 3	1 2<br>1 2 3	1 2<br>1 2 3
	C. Did your partner or any other partner ever force you to do something sexual that you found degrading or humiliating?	1 2<br>1 2 3	1 2<br>1 2 3	1 2<br>1 2 3
407	Check to see if women answered 'Yes' to any of the emotional violence questions (**see question 404**).	Emotional violence…………..1 No Emotional Violence……….2<br>↓<br>**1.** Before pregnancy " **If 1 and 2"→410**<br>2. During pregnancy<br>**3.** After pregnancy" **If 2 and 3"→411**		

408	Check to see if women answered 'Yes' to any of the physical violence questions (**see question 405**).	Physical violence…………..1 Physical Violence……….2<br>↓<br>**1.** Before pregnancy**'' If 1 and 2''→410**<br>2. During pregnancy<br>**3.** After pregnancy**'' If 2 and 3''→411**
409	Check to see if women answered 'Yes' to any of the sexual violence questions (**see question 406**).	Sexual violence…………..1 Sexual Violence……….2<br>↓<br>1. Before pregnancy **'' If 1 and 2''→410**<br>2. During pregnancy<br>3. After pregnancy **'' If 2 and 3''→411**
410	How you level violence occurrence during pregnancy as compared to pre-pregnancy period	Increased………1 Remained consistent ……2 Increased………..3<br>Don't remember………4 not agreed to answer…………5
411	How you level violence occurrence during pregnancy and postpartum	Increased………1 Remained consistent ……2 Increased………..3<br>Don't remember………4 not agreed to answer…………5

I have completed my interview thank you very much!
